# Comparisons of treatment satisfaction and health-related quality of life in patients with rheumatoid arthritis treated with tofacitinib and adalimumab

**DOI:** 10.1186/s13075-023-03047-1

**Published:** 2023-04-27

**Authors:** Seong-Kyu Kim, Sang-Heon Lee, Jiyu Sun, Soo Hyun Lee, Ja-Young Jeon, Hyun-Jeong Yoo, Jung-Yoon Choe

**Affiliations:** 1 Division of Rheumatology, Department of Internal Medicine, Daegu Catholic University School of Medicine, 33, Duryugongwon-Ro 17-Gil, Nam-Gu, Daegu, 42472 Republic of Korea; 2 Division of Rheumatology, Department of Internal Medicine, Research Institute of Medical Science, Konkuk University Medical Center, Konkuk University School of Medicine, Seoul, Republic of Korea; 3 Integrated Biostatistics Branch, Division of Cancer Data Science, National Cancer Center, Goyang-Si, Republic of Korea; 4 Division of Medical, Pfizer Biopharmaceuticals Group, Pfizer Pharmaceuticals Korea Limited, Seoul, Republic of Korea

**Keywords:** Rheumatoid arthritis, Treatment satisfaction, Quality of life, Tofacitinib, Adalimumab

## Abstract

**Background:**

As significant advances in the field of treatment for rheumatoid arthritis (RA), there is a great need to identify the healthcare outcomes such as treatment satisfaction and health-related quality of life (HRQoL) of patients with various treatment options. This study aims to identify the difference in the treatment satisfaction and HRQoL of patients with RA using different treatment options, by comparing the treatment satisfaction and HRQoL in patients with RA treated with tofacitinib and adalimumab in real-world settings in Korea, using propensity score methods.

**Methods:**

In this non-interventional, multicenter, cross-sectional study (NCT03703817), a total of 410 patients with RA diagnosis were recruited in 21 university-based hospitals throughout Korea. The treatment satisfaction and HRQoL were assessed using the Treatment Satisfaction Questionnaire for Medication (TSQM) and EQ-5D questionnaires self-reported by the patients. This study compared outcomes between two drug groups in unweighted, greedy matching, and stabilized inverse probability of treatment weight (IPTW) samples using propensity score.

**Results:**

In all three samples, tofacitinib group showed higher convenience domain of TSQM than that in the adalimumab group, but not effectiveness, side effects, and global satisfaction domains. Multivariable analysis using the covariates of demographic and clinical characteristics of the participants also showed consistent results in TSQM. No statistical difference in EQ-5D-based HRQoL was identified between two drug groups in all three samples.

**Conclusions:**

This study identified that tofacitinib shows higher treatment satisfaction in the convenience domain of TSQM rather than adalimumab, suggesting that various factors such as drug formulation, route or frequency of administration, and storage can have an impact on the treatment satisfaction, especially the convenience domain. These findings may be useful to patients and physicians when determining treatment options.

**Trial registration:**

ClinicalTrials.gov, NCT03703817.

**Supplementary Information:**

The online version contains supplementary material available at 10.1186/s13075-023-03047-1.

## Background

Rheumatoid arthritis (RA) is a systemic, chronic, inflammatory autoimmune disease that results in the joint deformity, physical disability, poor health-related quality of life (HRQoL), and increased mortality [1]. The advent of biological disease-modifying antirheumatic drugs (bDMARDs) and targeted synthetic DMARDs (tsDMARDs) has contributed significantly to the achievement of lower disease activity or remission for patients who showed insufficient treatment response or no drug tolerance to conventional synthetic DMARDs (csDMARDs) as their primary treatment in RA [2]. Optimal disease activity control has been found to affect not only the improvement of the patient’s symptoms, but also increased treatment satisfaction to medication and the improvement of the HRQoL in RA [3, 4].

Despite significant advances in the field of treatment for RA, there was a limit to satisfying patient’s unmet medical need for health care outcomes such as treatment satisfaction and HRQoL. Treatment satisfaction in RA was associated with multifactorial components, including disease activity, treatment cost, physical function capacity, or pain [5, 6]. Additionally, many evidence have been suggested that the treatment satisfaction of patients with RA might be different greatly by the type of DMARDs (bDMARDs vs. csDMARDs) or route of administration in bDMARDs (intravenous vs. subcutaneous or oral vs. parenteral) [3, 6, 7].

With the advent of tsDMARDs such as tofacitinib, they have been emerging as a new therapeutic option for the treatment of RA. Several studies including randomized controlled trials have shown that tofacitinib has safety and efficacy profile equivalent to adalimumab, one of the most widely used bDMARDs [8, 9]. However, only few studies have investigated the treatment satisfaction and HRQoL of tofacitinib in patients with RA [10, 11] while those of adalimumab has been investigated in many studies [12, 13]. Analysis from MUSICA Trial showed improvement of the treatment satisfaction and HRQoL of adalimumab with methotrexate (MTX) combination to only MTX use [14]. There is a study that has investigated the HRQoL of both tofacitinib and adalimumab in patients with RA. In this study, tofacitinib with MTX combination for the treatment of RA had greater improvement of HRQoL than adalimumab with MTX, with at least similar efficacy to adalimumab [15].

In Korea, the treatment satisfaction and HRQoL of patients with RA, especially using bDMARDs or tofacitinib, have not been identified. There is an increasing interest in the treatment satisfaction and HRQoL of patients with RA using tofacitinib in real-world clinical practices as the reimbursement coverage for tofacitinib has expanded widely since 2017. And it is important to evaluate the treatment satisfaction and HRQoL of patients in management of chronic disease that are strongly affected by various factors of treatment environment in real-world, using reliable statistical models. Therefore, this study was conducted to identify the difference in the treatment satisfaction and HRQoL of patients with RA using different treatment options, by comparing the treatment satisfaction and HRQoL in patients with RA treated with tofacitinib and adalimumab in real-world settings in Korea, using propensity score methods.

## Methods

### Study population

This non-interventional, multicenter, cross-sectional study enrolled patients with RA diagnosed according to the 2010 ACR/EULAR classification criteria [16] from June 2018 to March 2020 at 21 university-based institutions nationwide in Korea. The patients who met the inclusion or exclusion criteria were enrolled in this study. Briefly, inclusion criteria were as follows: patients aged ≥ 19 years with patients who were taking tofacitinib or adalimumab for ≥ 6 months. Further, the following patients were excluded: patients who were taking tofacitinib or adalimumab for ≥ 2 years (to reduce the difference in the duration of current treatment among enrolled patients, which could have resulted from the differing start dates of reimbursement expansion for the two drugs), patients who were taking azathioprine and cyclosporine in combination (because tofacitinib should not be used in combination with potent immunosuppressants, such as azathioprine and cyclosporine), and patients who were taking bDMARDs for diseases other than RA.

Among a total of 411 RA patients enrolled in this study, 281 patients were included in the tofacitinib group and 130 patients in the adalimumab group in the unweighted sample (see figure, Additional file 1). The final participants included in the analyses were 410 RA patients, excluding one patient in the adalimumab group with two or more missing data in outcome variables. As a result of propensity score matching method by applying a 2:1 matching ratio for both tofacitinib and adalimumab treatment groups, a total of 231 patients were selected for the greedy matching sample: 139 and 92 patients in the tofacitinib and adalimumab groups, respectively. Lastly, as regards propensity score weighting method, stabilized inverse probability of treatment weight (IPTW), a total of 325 participants were identified, including 226 and 99 participants classified into the tofacitinib and adalimumab groups, respectively.

The protocol of this study was approved by the Institutional Review Board of each institute at all institutes participated in this study. All participants voluntarily signed written consent forms. This study was registered with ClinicalTrials.gov (NCT03703817).

### Data collection

Age and sex as demographic characteristics were identified at the time of enrollment. Education level was classified into less than high school and college or more. Annual household income was divided into less than 50 million South Korean won and more than 50 million won. Employment status was divided into employment and unemployment. Body mass index was calculated by dividing body weight (kg) and height in meters squared (m^2^). Charlson Comorbidity Index score was identified. Disease duration and duration of current treatment were measured in months. It was confirmed whether concomitant use of methotrexate, nonsteroidal anti-inflammatory drugs (NSAIDs), and steroid was administered. In addition, methotrexate dosage was also determined. Disease activity was assessed by C-reactive protein (CRP) or erythrocyte sedimentation rate (ESR) with tender joint count, swollen joint count, and general health on a 100-mm visual analog scale (VAS) (0 = best, 100 = worst), in two disease activity scores of 28 joints (DAS28-CRP and DAS28-ESR), respectively [17].

### Measurement of clinical outcomes

The treatment satisfaction and HRQoL were assessed using the Treatment Satisfaction Questionnaire for Medication (TSQM) [18] and the EuroQoL-5 dimension (EQ-5D) [19] questionnaires self-reported by the patients. The TSQM Version 1.4 was used in this study, consisting of 14 questions and four domains: effectiveness, convenience, side effects, and global satisfaction. The EQ-5D contained a descriptive system (5 questions) in three level, EQ-5D, as well as the EuroQoL-VAS (EQ-VAS) from 0 (worst) to 100 (best). This study used the EQ-5D approved for the validity and reliability for measuring HRQoL in the Korean population [20].

### Statistical analysis

This study compared differences in treatment satisfaction and HRQoL between two drug groups in three samples. All patients were included in the unweighted sample and the patients in other samples were selected using the propensity score (PS) with two balancing methods. Propensity score matching (PSM) and inverse probability treatment weighting (IPTW) were performed to minimize the bias of confounding factors and facilitate the comparability between groups. The PS was calculated using the covariates of demographic and clinical characteristics of the participants, except DAS28 components. Greedy matching and stabilized IPTW methods were used to balance the covariates. For greedy matching, the matching ratio was set as 2:1 to reflect the number of patients in the tofacitinib and adalimumab groups, with 0.25 × standard deviation (SD) of the PS for the caliper. The stabilized IPTW created a pseudo-dataset with PS and preserves the sample size of the original data.

The baseline characteristics were reported as original unweighted sample, matched sample, and weighted sample to confirm the balance in covariates of the two groups before and after PS adjustment. Balance in characteristics between two groups was assessed using the standardized mean difference (SMD). Imbalanced was defined as a SMD of > 0.1. To compare the clinical outcomes of two groups in univariable and multivariable analysis, we confirmed the distribution of outcome variables (Additional file 2). Since the distribution of EQ-5D is strongly left skewed, we created a binary outcome variable generated by dividing into two categories based on the median of EQ-5D. And for the side effect, since more than 75% of the sample scores 100, we use a binary outcome variable of whether the score is 100 or not. Generalized Estimating Equation (GEE) method was used in the greedy matching sample and weighted linear regression, weighted generalized linear regression, and weighted ordinal logistic regression models were performed in the stabilized IPTW sample. For comparing outcomes for the treatment satisfaction and HRQoL between two drug groups in univariable and multivariable analysis, logistic regression and linear regression models were used for the binary outcome variables and the continuous outcomes variables in the unweighted sample, respectively. In the greedy matching sample, outcomes were investigated using conditional logistic regression and linear mixed models, whereas weighted logistic regression and weighted linear regression models were used in the stabilized IPTW sample. Multivariable analyses with these models were performed to compare outcomes by adjusting all variables for demographic and clinical characteristics as confounders. Because covariates imbalance can be remained even after PS matching or applying IPTW method, we conducted the multivariable analysis according to adjustment of confounders. Statistical analysis was conducted using SAS (version 9.4; SAS Institute) and R software (version 3.6.2; R Foundation).

## Results

### General characteristics of study population

The demographical and clinical characteristics of patients treated with tofacitinib (*n* = 281) and adalimumab (*n* = 129) in three samples are shown in Tables 1 and 2. In the unweighted sample, the average age of the total study participants was 53.5 years and significantly different in both the tofacitinib (54.5 years) and adalimumab groups (51.5 years) (SMD = 0.249). Methotrexate was concomitantly used to 75.6% and 95.2% of the participants from the tofacitinib and adalimumab groups, respectively, and concomitant methotrexate usage ratio was statistically significantly different in both groups (SMD = 0.579). However, other demographical and clinical characteristics between the two groups were not noted in the unweighted sample. In addition, no significant difference was observed in the demographical and clinical characteristics between the two groups in both samples by greedy matching with PS and by stabilized IPTW.

**Table 1:** Demographic characteristics of patients in unweighted, Greedy-matching, and stabilized inverse probability of treatment–weighed samples

Characteristics (mean ± SD or *n*(%))	Total (*n* = 410)	Unweighted			Greedy matching			Stabilized IPTW		
		**Tofacitinib**	**Adalimumab**	**SMD**	**Tofacitinib**	**Adalimumab**	**SMD**	**Tofacitinib**	**Adalimumab**	**SMD**
		**(*n* = 281)**	**(*n* = 129)**		**(*n* = 139)**	**(*n* = 92)**		**(*n* = 226)**	**(*n* = 99)**	
**Age**	53.52 ± 12.19	54.47 ± 12.10	51.45 ± 12.18	0.249	52.73 ± 11.81	51.32 ± 11.50	0.121	53.56 ± 12.48	53.27 ± 11.35	0.025
**Sex**										
Male	54 (13.17)	40 (14.24)	14 (10.85)	0.102	16 (11.51)	8 (8.70)	0.094	28 (12.49)	15 (15.44)	0.085
Female	356 (86.83)	241 (85.77)	115 (89.15)		123 (88.49)	84 (91.30)		197 (87.51)	82 (84.56)	
**Education**										
≤ high school	279 (67.89)	194 (69.04)	85 (65.89)	0.067	94 (67.63)	61 (66.3)	0.028	153 (67.98)	65 (67.55)	0.009
College or more ≤	131 (32.11)	87 (30.96)	44 (34.11)		45 (32.37)	31 (33.7)		72 (32.03)	31 (32.45)	
**House annual income**^a^** (10,000 won)**^**b**^										
< 5000	277 (67.89)	191 (68.46)	86 (66.67)	0.038	94 (67.63)	66 (71.74)	0.090	153 (67.72)	69 (71.26)	0.077
5000 ≤	131 (32.11)	88 (31.54)	43 (33.33)		45 (32.37)	26 (28.26)		73 (32.28)	28 (28.74)	
**Employment**										
Employed	178 (45.64)	122 (46.04)	56 (44.8)	0.025	67 (48.2)	45 (48.91)	0.014	104 (45.89)	47 (48.41)	0.050
Unemployed	212 (54.36)	143 (53.96)	69 (55.2)		72 (51.8)	47 (51.09)		122 (54.11)	50 (51.59)	
**BMI**	23.43 ± 3.56	23.56 ± 3.54	23.13 ± 3.60	0.118	23.55 ± 3.52	23.30 ± 3.78	0.068	23.57 ± 3.58	23.56 ± 3.89	0.001

^a^2 missing for house annual income

^b^10,000 won = 8.24 USD

*IPTW* inverse probability of treatment weighting, *SD* standard deviation, *n* number, *SMD* standardized mean differences, *won*, South Korean Won, *BMI* body mass index

**Table 2:** Clinical characteristics of patients in unweighted, greedy-matching, and stabilized inverse probability of treatment–weighed samples

Characteristics (mean ± SD or *n*(%))	Total (*n* = 410)	Unweighted			Greedy matching			Stabilized IPTW		
		**Tofacitinib**	**Adalimumab**	**SMD**	**Tofacitinib**	**Adalimumab**	**SMD**	**Tofacitinib**	**Adalimumab**	**SMD**
		**(*n* = 281)**	**(*n* = 129)**		**(*n* = 139)**	**(*n* = 92)**		**(*n* = 226)**	**(*n* = 99)**	
**CCI score**										
0	339 (82.68)	229 (81.5)	110 (85.27)	0.108	119 (85.61)	79 (85.87)	0.062	187 (82.76)	80 (83.04)	0.068
1	58 (14.15)	42 (14.95)	16 (12.4)		18 (12.95)	11 (11.96)		33 (14.52)	15 (15.22)	
2 ≤	13 (3.17)	10 (3.56)	3 (2.33)		2 (1.44)	2 (2.17)		6 (2.72)	2 (1.74)	
**Disease duration (months)**	67.38 ± 70.13	71.32 ± 70.52	58.87 ± 68.79	0.179	59.15 ± 62.59	61.05 ± 71.87	0.028	70.72 ± 70.00	67.92 ± 80.92	0.037
**Duration of current treatment (months)**	11.33 ± 5.11	11.23 ± 4.93	11.55 ± 5.49	0.061	11.07 ± 4.97	11.25 ± 5.34	0.035	11.26 ± 4.99	11.19 ± 5.25	0.014
**Concomitant use of MTX**										
Yes	331 (81.73)	211 (75.63)	120 (95.24)	0.579	128 (92.09)	87 (94.57)	0.099	180 (79.94)	81 (83.27)	0.086
No	74 (18.27)	68 (24.37)	6 (4.76)		11 (7.91)	5 (5.44)		45 (20.06)	16 (16.73)	
**MTX dose (1000 mg)**	2.07 ± 2.81	1.96 ± 2.82	2.31 ± 2.78	0.124	2.26 ± 3.09	2.35 ± 2.66	0.030	2.09 ± 2.99	2.37 ± 2.97	0.095
**Concomitant use of NSAIDs**										
Yes	289 (70.49)	200 (71.17)	89 (68.99)	0.048	104 (74.82)	67 (72.83)	0.045	163 (72.17)	70 (72.05)	0.003
No	121 (29.51)	81 (28.83)	40 (31.01)		35 (25.18)	25 (27.17)		63 (27.84)	27 (27.95)	
**Concomitant use of steroid**										
Yes	289 (70.49)	202 (71.89)	87 (67.44)	0.097	99 (71.22)	61 (66.3)	0.106	156 (69.34)	67 (69.12)	0.005
No	121 (29.51)	79 (28.11)	42 (32.56)		40 (28.78)	31 (33.7)		69 (30.66)	30 (30.88)	
**DAS28-CRP**	2.67 ± 1.17	2.65 ± 1.16	2.71 ± 1.19	0.052	2.67 ± 1.16	2.768 ± 1.212	0.082	2.705 ± 1.233	2.826 ± 1.348	0.094
**DAS28-ESR**	3.33 ± 1.30	3.35 ± 1.26	3.30 ± 1.39	0.032	3.31 ± 1.17	3.320 ± 1.396	0.010	3.380 ± 1.297	3.434 ± 1.551	0.038
**DAS28 components**										
ESR (mm/h)	24.63 ± 20.06	25.07 ± 18.93	23.69 ± 22.37	0.066	22.66 ± 15.25	23.17 ± 20.81	0.028	25.17 ± 18.36	27.48 ± 25.81	0.103
CRP	4.00 ± 9.27	3.97 ± 9.77	4.07 ± 8.12	0.012	4.31 ± 12.72	4.81 ± 9.20	0.045	4.76 ± 13.58	6.22 ± 10.09	0.123
Tender joint count	2.74 ± 3.95	2.58 ± 3.84	3.09 ± 4.18	0.126	2.73 ± 4.00	3.11 ± 4.13	0.092	2.88 ± 4.36	3.12 ± 4.72	0.051
Swollen joint count	1.909 ± 3.832	1.793 ± 3.721	2.164 ± 4.068	0.095	1.892 ± 3.812	2.21 ± 4.02	0.080	2.13 ± 4.33	2.41 ± 4.68	0.061
GH VAS	28.96 ± 20.62	29.93 ± 21.15	26.89 ± 19.37	0.150	29.07 ± 20.88	27.32 ± 19.53	0.086	29.00 ± 21.11	29.68 ± 20.58	0.033

*IPTW* inverse probability of treatment weighting, *SD* standard deviation, *n* number, *SMD* standardized mean differences, *CCI score* Charlson Comorbidity Index score, *MTX* methotrexate, *NSAIDs* nonsteroidal anti-inflammatory drugs, *DAS* Disease Activity Score, *CRP* C-reactive protein level, *ESR* erythrocyte sedimentation rate, *GH* general health, *VAS* visual analog scale

### Comparison of treatment satisfaction and HRQoL between tofacitinib and adalimumab

The average scores of convenience domain of TSQM were higher in the tofacitinib (69.87 (standard deviation (SD): 13.37), 68.79 (13.35), and 69.55 (13.62), respectively) than that in the adalimumab group (63.76 (13.15), 64.95 (12.85), and 63.96 (11.86), respectively), but not effectiveness, side effects, and global satisfaction domains of TSQM in all three samples (Table 3). In Additional file 3, the ranges of four domains of TSQM were shown in all three samples. In the comparison of HRQoL between tofacitinib and adalimumab, no statistical difference was found in HRQoL outcomes, including EQ-VAS and EQ-5D, between tofacitinib and adalimumab groups in all three samples.

**Table 3:** Patient outcomes in unweighted, greedy-matching, and stabilized inverse probability of treatment–weighed samples

Outcomes	Total (*n* = 410)	Unweighted sample			Greedy matching			Stabilized IPTW		
		**Tofacitinib**	**Adalimumab**	**Difference or log odds ratio** **(95%CI)**	**Tofacitinib**	**Adalimumab**	**Difference or log odds ratio** **(95%CI)**	**Tofacitinib**	**Adalimumab**	**Difference or log odds ratio** **(95%CI)**
		**(*n* = 281)**	**(*n* = 129)**		**(*n* = 139)**	**(*n* = 92)**		**(*n* = 226)**	**(*n* = 99)**	
**TSQM**										
**Effectiveness**	63.08 ± 13.75	62.40 ± 13.77	64.58 ± 13.64	− 2.19 (− 5.06, 0.69)	62.27 ± 13.30	64.19 ± 13.32	− 1.88 (− 5.23, 1.48)	62.09 ± 13.91	62.25 ± 12.69	− 0.16 (− 3.25, 2.93)
**Side effects**^b^**,** *n* (%)	356 (86.8)	241 (85.8)	115 (89.1)	− 0.31 (− 0.96, 0.34)^a^	115 (82.7)	82 (89.1)	− 0.67 (− 0.15, 0.20)^a^	93.95 ± 16.25	96.91 ± 11.44	− 0.63 (− 1.42, 0.17) ^a^
**Convenience**	67.95 ± 13.58	69.87 ± 13.37	63.76 ± 13.15	6.11 (3.34, 8.88)	68.79 ± 13.35	64.95 ± 12.85	3.84 (0.38, 7.30)	69.55 ± 13.62	63.96 ± 11.86	5.59 (2.66, 8.53)
**Global satisfaction**	58.24 ± 16.14	57.19 ± 16.46	60.52 ± 15.24	− 3.33 (− 6.60, − 0.06)	56.68 ± 15.41	59.86 ± 15.56	− 3.17 (− 7.18, 0.84)	57.04 ± 16.16	58.72 ± 14.68	− 1.68 (− 5.26, 1.90)
**EQ-VAS**	65.42 ± 19.41	64.86 ± 19.63	66.78 ± 19.02	− 1.92 (− 5.95, 2.11)	65.40 ± 19.97	65.67 ± 19.55	− 0.27 (− 5.48, 4.95)	65.01 ± 19.18	67.59 ± 17.73	− 2.58 (− 6.90, 1.73)
**EQ-5D**^**c**^**,** *n* (%)	206 (50.5)	147 (52.3)	59 (46.5)	0.23 (− 0.18, 0.65)^a^	72 (51.8)	41 (44.6)	0.31 (− 0.24, 0.87) ^a^	0.81 ± 0.17	0.80 ± 0.14	0.24 (− 0.24, 0.72)^a^

^a^Log odds ratio

^b^Whether TSQM (side effects) is 100 or not

^c^EQ-5D < median or EQ-5D ≥ median

*IPTW* inverse probability of treatment weighting, *SD* standard deviation, *n* number, *CI* confidence interval, *TSQM* Treatment Satisfaction Questionnaire for Medication, *EQ* EuroQol, *VAS* visual analog scale, *EQ-5D* EuroQol-5-Dimension

### Association of treatment satisfaction and HRQoL in tofacitinib compared to adalimumab

The results from multivariable analysis with adjusting all variables for demographic and clinical characteristics showed that tofacitinib treatment was positively associated with the convenience domain of TSQM, compared to adalimumab treatment in all three samples (95% confidence interval of coefficient: (1.127, 7.807), (0.290, 7.236), and (2.149, 8.363), respectively). However, there were no association of effectiveness, side effects, and global satisfaction domains of TSQM, EQ-VAS, and EQ-5D between tofacitinib and adalimumab in three samples, with controlling the confounder effects of demographic and clinical characteristics (Table 4).

**Table 4:** Multivariable analysis of outcomes in unweighted, greedy-matching, and stabilized inverse probability of treatment–weighed samples

Outcomes	Unweighted sample		Greedy matching		Stabilized IPTW	
	**(*n* = 410)**		**(*n* = 231)**		**(*n* = 325)**	
	**Coef.**^a^	**95%CI of Coef**	**Coef.**^a^	**95%CI of Coef**	**Coef.**^a^	**95%CI of Coef**
**TSQM**						
**Effectiveness**	− 2.255	(− 5.728, 1.218)	− 1.557	(− 4.914, 1.800)	− 0.18	(− 3.398, 3.038)
**Side effects**^b^	− 0.642	(− 1.45, 0.166)	− 2.184	(− 5.081, 0.713)	− 0.655	(− 1.466, 0.156)
**Convenience**	4.467	(1.127, 7.807)	3.763	(0.290, 7.236)	5.256	(2.149, 8.363)
**Global satisfaction**	− 3.129	(− 7.169, 0.911)	− 2.977	(− 7.062, 1.108)	− 1.683	(− 5.421, 2.055)
**EQ-VAS**	− 0.656	(− 5.581, 4.269)	0.027	(− 5.345, 5.399)	− 2.661	(− 7.200, 1.878)
**EQ-5D**^c^	0.191	(− 0.370, 0.752)	0.540	(− 0.215, 1.295)^*(e)*^	0.246	(− 0.293, 0.785)^*(h)*^

^a^The difference in outcomes between tofacitinib and adalimumab (a reference) estimated by multivariable analyses with performed by adjusting all variables for demographic and clinical characteristics, such as age, sex, CCI score, and disease duration

^b^Whether TSQM (side effects) is 100 or not

^c^EQ-5D < median or EQ-5D ≥ median

*IPTW* inverse probability of treatment weighting, *n* number, *Coef.* coefficient, *CI* confidence interval, *TSQM* Treatment Satisfaction Questionnaire for Medication, *EQ* EuroQol, *VAS* visual analog scale, *EQ-5D* EuroQol-5-Dimension

## Discussion

Identifying the treatment satisfaction of patients to medication has been considered as a crucial component to lead to improvement of clinical outcome [21]. And HRQoL is also an important outcome in chronic disease management, such as RA which cause painful joints and functional disability, leading to poor physical and psychological HRQoL [22, 23]. This study investigated and compared the treatment satisfaction and HRQoL of patients with RA treated with tofacitinib and adalimumab in real-world settings in Korea, to identify the difference in the treatment satisfaction and HRQoL of patients with RA using different treatment options. The results of this study showed there were no statistical difference in effectiveness, side effects, and global satisfaction domains of TSQM for the treatment satisfaction and EQ-5D-based HRQoL between two drug groups in all three samples. However, this study found that the convenience domain of TSQM in the tofacitinib group had higher sub-scores than that in adalimumab group. The differences in average scores of convenience domain of TSQM between tofacitinib and adalimumab groups were 6.11, 3.84, and 5.59 in three samples, respectively.

The factors that affect the treatment satisfaction of patients are very diverse, including not only by route or frequency of administration and storage but also the patient’s characteristics such as age, disease severity, and social activity. Treatment satisfaction in RA was found to be associated with various factors such as reduction of inflammation and pain, improvement of functional status, disease severity, and seropositivity [5, 6]. It was also reported that treatment satisfaction was tightly linked with medication preference, compliance, and adherence [21]. Treatment satisfaction based on the TSQM was associated with the route of administration (oral, injectable, or topical) [18]. Several patient considerations for subcutaneous injections may also be relevant. Self-injection of subcutaneous agents requires training and support by a healthcare professional and requires refrigeration. In addition, self-injection may be difficult in patients with functional impairment and limitation in hand movement, which may affect treatment adherence [24, 25]. A population-based study in China found that satisfaction levels in convenience domain of TSQM-II with all medications were higher than those with bDMARDs [81.3 (18.6–100) vs. 75 (0–100)], without no differences of effectiveness and side effects domains [6]. However, the results from a subgroup analysis of patients with bDMARDs or tsDMARDs in the SENSE study showed that global and effectiveness sub-scores of patients with an oral DMARD were lower than those of parenteral DMARD [3] while the most patients of Japanese subpopulation preferred oral route of administration (60.7%) [7]. Several evidence suggest that the routes of administration can affect patient satisfaction with treatment in RA. The results of this study also suggested that the oral route of administration of tofacitinib may be a factor that affects the treatment satisfaction, compared with that of adalimumab. However, the treatment satisfaction such as convenience domain must be balanced with other factors related to medical administration although there are many consequences for the route of administration. Patients with more severe disease may place less value on mode and frequency of administration and prioritize improvements in pain and function [26].

Treatment satisfaction of patients with RA using TSQM have been investigated with varying results in many studies. The average TSQM score in this study on 410 study participants including 281 and 129 patients in the tofacitinib and adalimumab groups, respectively, was 63.1 in the effectiveness domain, 95.1 in the side effect domain, and 68.0 in the convenience domain, and 58.2 in the global satisfaction domain. While approximately 90% of the 258 participants had experienced using biologic DMARDs or were taking biologic DMARDs, a study in USA reported that TSQM scores were 59, 59, 72, and 65 for effectiveness, side effects, convenience, and global satisfaction, respectively [4]. In China, medians of TSQM-II sub-scores of patients treated with bDMARDs in China had global satisfaction with TSQM-II scores of 83.3 and 75.0, respectively [6]. As patient-reported outcomes are based on patients’ subjective experiences and perceptions, and these experiences can be influenced by various factors such as age, gender, disease severity, cultural background, and treatment context, the TSQM scores may differ depending on the condition being studied or the population being treated. However, using a valid and reliable measure of TSQM could allow relative comparisons of treatment satisfaction across medication types, patient conditions, and countries [18]. In this study, patients who received tofacitinib were more likely to report higher levels of satisfaction with treatment convenience compared to those who received adalimumab. In other words, higher scores in the convenience domain of the TSQM suggest that patients found the treatment more convenient and were more satisfied with this aspect of their treatment experience. The TSQM can also provide valuable insights, such as overall satisfaction with treatment, predictors of treatment satisfaction, and satisfaction with specific aspects of treatment, into patient satisfaction with RA treatment, helping healthcare professionals to better understand and address patients’ needs and concerns. The results of our study, as well as previous studies, using TSQM as a general measure of treatment satisfaction across various treatment settings, can provide clinically meaningful and valuable evidence to support treatment decision-making by patients and physicians in real-world clinical practices.

EQ-5D, one of the most used HRQoL tools in the field of clinical research developed by the EuroQoL group, was used to measure HRQoL of participants in this study [19]. In the 6th month, phase 3, randomized, placebo-controlled trial, tofacitinib monotherapy led to improvement of HRQoL assessed by the Medical Outcomes Survey Short Form-36 [27]. In addition, adalimumab plus methotrexate also improved the HRQoL compared to methotrexate monotherapy [28]. Furthermore, tofacitinib plus methotrexate and adalimumab plus methotrexate showed meaningful benefit of improvement of HRQoL in ORAL Strategy [21]. Moreover, this study found no statistical difference in EQ-5D of between tofacitinib and adalimumab, which was consistent with that of the previous study [29]. However, other factors, including age, sex, employment, body mass index, concomitant use of NSAIDs or steroid, or disease activity, were found to be associated with EQ-5D. The means of EQ-5D or EQ-VAS of study population vary across countries. In this study, the mean of EQ-5D of total subjects was 0.82, whereas pooled EQ-5D of meta-analysis for 31 studies conducted in Asia was 0.66 [30]. The present study found that higher EQ-5D score was associated with male, younger ages, and lower disease activity, which are consistent with the results of previous studies [30, 31].

This study had some limitations. First, this study was an observational study that has limitations in inferring correlations owing to the absence of randomization, considering selection bias. In this study, two statistical methods with PS were used to balance between the drug groups. The results of comparison of outcomes between tofacitinib and adalimumab groups were robust in three samples (unweighted, greedy-matching, and stabilized inverse probability of treatment–weighed samples). However, although the PS can reduce selection bias between different patient groups, there still may be confounders affecting outcomes [32, 33]. Second, treatment satisfaction is difficult to be defined and evaluated because it can include patient’s satisfaction about various treatment experiences, from a drug to the health care delivery system [34]. Moreover, various factors affect treatment satisfaction, and the associations are complicated [8, 21]. Similar to treatment satisfaction, several different factors also affect HRQoL [30, 31]. Therefore, this study could have limitations due to these difficulties although TSQM and EQ-5D are widely authorized and frequently used tools [15, 16, 19, 35]. Third, this study used a self-reported questionnaire; there may be individual differences in understanding of each question. Lastly, since this study was conducted in a single country, it may be difficult to generalize cultural and geographical influences such as access and health care environment.

Notwithstanding these limitations, this study has remarkable strengths. This study is a multi-center study of 21 university hospitals in Korea, and it is a real-world data that enrolled 410 Korean patients with RA on a large scale. A variety of valid statistical methods were used to reduce various biases and confounding effects that are relatively hard to control in real-world setting. In addition, this study is a direct comparison study on the treatment satisfaction between tofacitinib and adalimumab and can provide valuable real-world evidence for determining treatment option as well as for understanding of RA patients deeply.

## Conclusions

The results of this study showed that, in all three comparison methods, treatment satisfaction in convenience domain of TSQM was higher in the tofacitinib group than that in adalimumab group. However, there was no difference between tofacitinib and adalimumab treatment in effectiveness, side effects, and global satisfaction domains of treatment satisfaction. The result of multivariable analysis also confirmed that treatment satisfaction in convenience domain of TSQM was higher in the tofacitinib group after adjusting many covariates. Tofacitinib and adalimumab have different characteristics, such as drug formulation, route or frequency of administration, and storage method, and these characteristics may have worked in combination to show differences in the convenience domain of treatment satisfaction between the two drugs. In addition, we found no difference of HRQoL based on EQ-5D between tofacitinib and adalimumab, although two drugs had positive effect of QoL on patients with RA [15, 27, 28]. Because patient’s perception for treatment satisfaction and assessment of HRQoL are crucial steps to improving clinical outcomes [21], further prospective studies are needed on various components that can determine treatment satisfaction, including the convenience, and affect HRQoL in patients with RA.

## Supplementary Information

**Additional file 1.** Participant flowchart.

**Additional file 2.** Histograms of patient outcomes in the unweighted sample.

**Additional file 3.** TSQM summary scores in (a) unweighted, (b) Greedy-matching, and (c) stabilized inverse probability of treatment–weighed samples.

## Data Availability

The data underlying this article cannot be shared publicly considering the privacy of the individuals involved in the study. However, the data can be shared upon reasonable request to the corresponding author.

## Abbreviations

CRP: C-reactive protein
bDMARDs: Biological disease-modifying antirheumatic drugs
ESR: Erythrocyte sedimentation rate
GEE: Generalized Estimating Equation
HRQoL: Health-related quality of life
IPTW: Inverse probability of treatment weight
NSAID: Nonsteroidal anti-inflammatory drug
PS: Propensity score
RA: Rheumatoid arthritis
SD: Standard deviation
TSQM: Treatment Satisfaction Questionnaire for Medication
VAS: Visual analog scale
