# Supplementary material for: Comparisons of treatment satisfaction and health-related quality of life in patients with rheumatoid arthritis treated with tofacitinib and adalimumab
Source: Arthritis Res Ther. 2023 Apr 27;25:68. doi: 10.1186/s13075-023-03047-1 (PMC10134656; doi:10.1186/s13075-023-03047-1)
Supplement: Supplementary file 1 — Additional file 1. Participant flowchart. [file 13075_2023_3047_MOESM1_ESM.docx]

Additional file 1. Participant flowchart
