# Supplementary figures and images for: Comparisons of treatment satisfaction and health-related quality of life in patients with rheumatoid arthritis treated with tofacitinib and adalimumab
Source: Arthritis Res Ther. 2023 Apr 27;25:68. doi: 10.1186/s13075-023-03047-1 (PMC10134656; doi:10.1186/s13075-023-03047-1)

Additional file 2. Histograms of patient outcomes in the unweighted sample


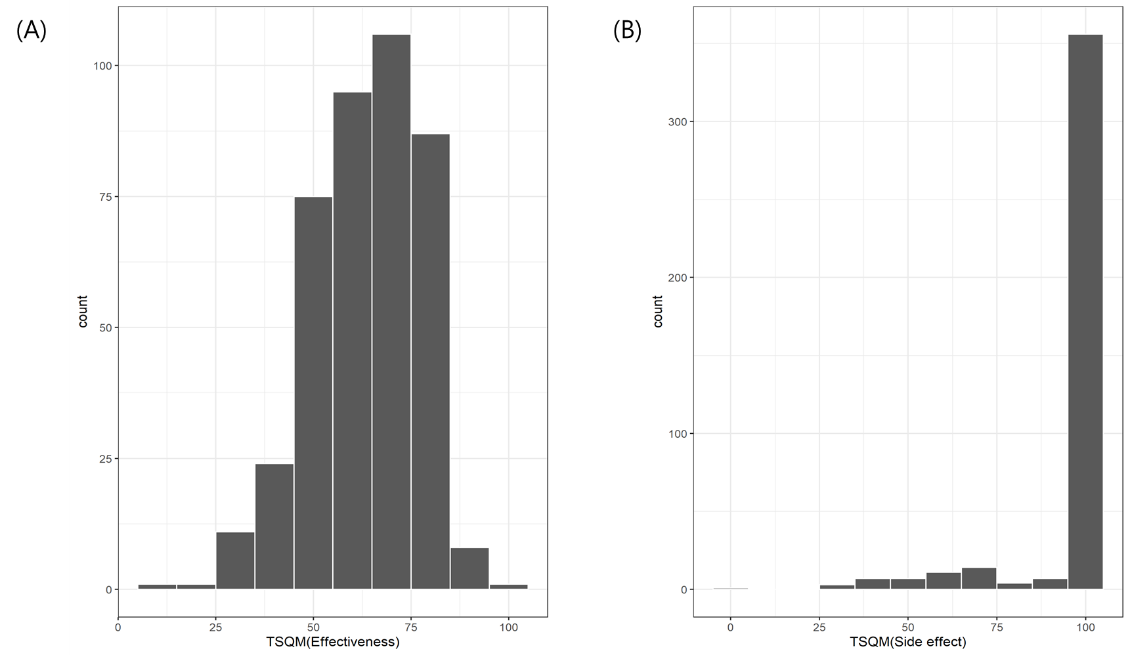


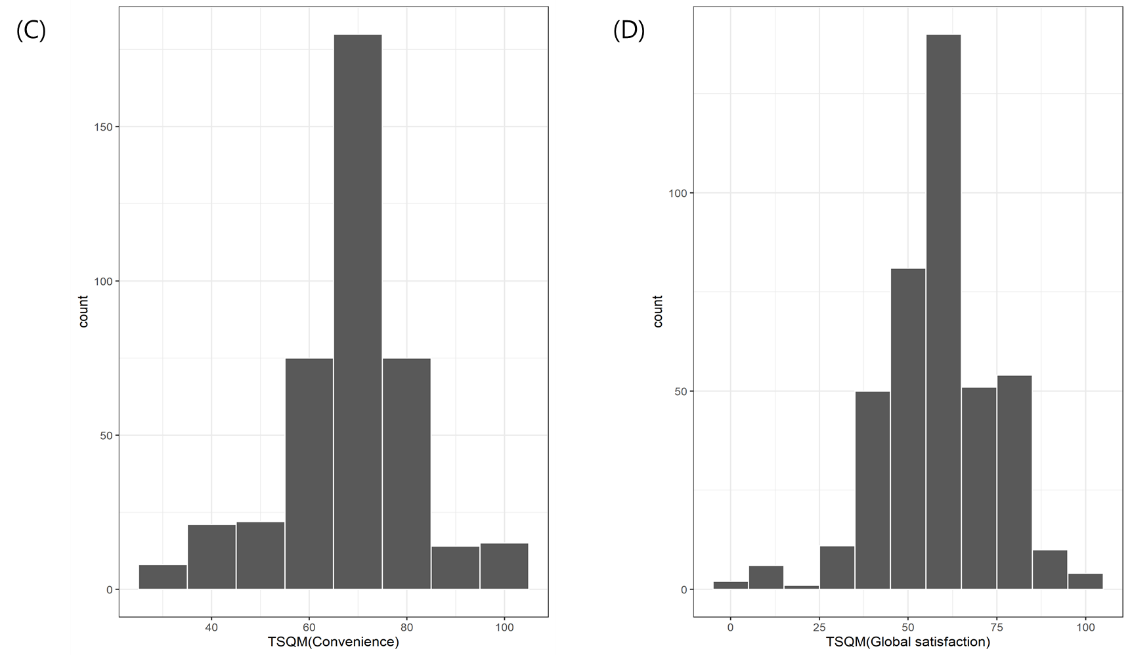


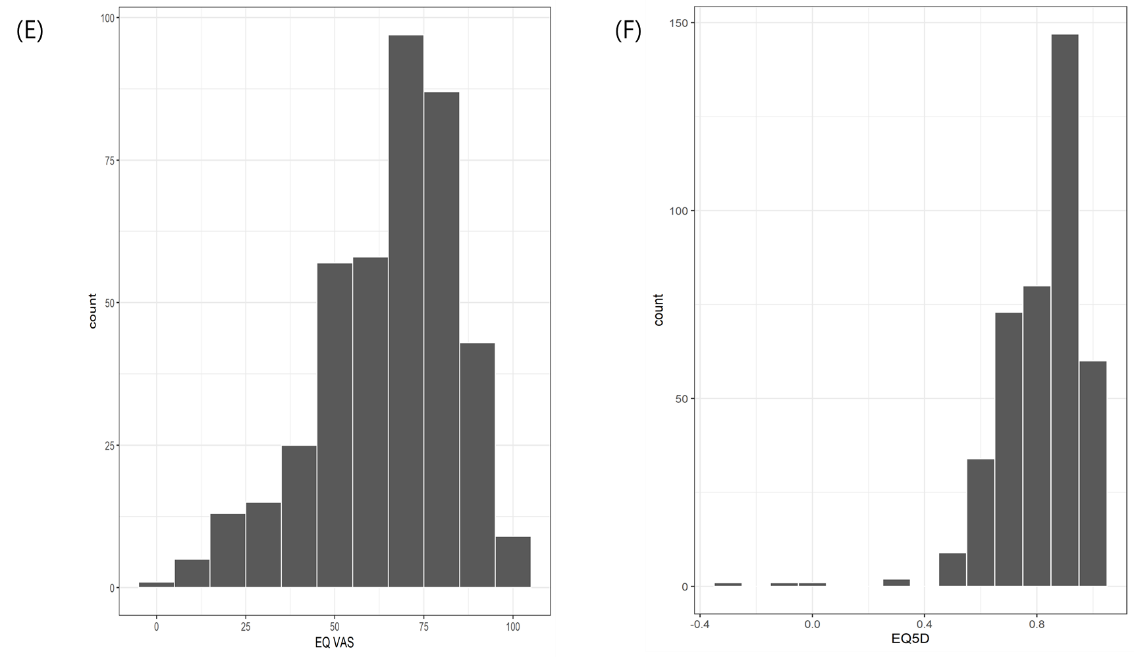

Supplement: Supplementary file 2 — Additional file 2. Histograms of patient outcomes in the unweighted sample. [file 13075_2023_3047_MOESM2_ESM.docx]
