# Supplementary material for: Comparisons of treatment satisfaction and health-related quality of life in patients with rheumatoid arthritis treated with tofacitinib and adalimumab
Source: Arthritis Res Ther. 2023 Apr 27;25:68. doi: 10.1186/s13075-023-03047-1 (PMC10134656; doi:10.1186/s13075-023-03047-1)
Supplement: Supplementary file 3 — Additional file 3. TSQM summary scores in (a) unweighted, (b) Greedy-matching, and (c) stabilized inverse probability of treatment–weighed samples. [file 13075_2023_3047_MOESM3_ESM.docx]

Additional file 3. TSQM summary scores in (a) unweighted, (b) Greedy-matching, and (c) stabilized inverse probability of treatment–weighed samples

| **(a) Unweighted sample (n = 410)** | | | | | | | |
| --- | --- | --- | --- | --- | --- | --- | --- |
|  | **mean** | **SD** | **min** | **Q1** | **median** | **Q3** | **max** |
| **Effectiveness** | 63.08 | 13.75 | 11.11 | 50.00 | 61.11 | 72.22 | 100.00 |
| **Side effects** | 95.06 | 14.46 | 0.00 | 100.00 | 100.00 | 100.00 | 100.00 |
| **Convenience** | 67.95 | 13.58 | 27.78 | 61.11 | 66.67 | 77.78 | 100.00 |
| **Global satisfaction** | 58.24 | 16.14 | 0.00 | 50.00 | 57.14 | 71.43 | 100.00 |
|  | | | | | | | |
| **(b) Greedy-matching sample (n = 231)** | | | | | | | |
|  | **mean** | **SD** | **min** | **Q1** | **median** | **Q3** | **max** |
| **Effectiveness** | 63.24 | 13.78 | 22.22 | 50.00 | 61.11 | 72.22 | 100.00 |
| **Side effects** | 94.99 | 14.73 | 0.00 | 100.00 | 100.00 | 100.00 | 100.00 |
| **Convenience** | 67.34 | 13.57 | 27.78 | 61.11 | 66.67 | 72.22 | 100.00 |
| **Global satisfaction** | 58.10 | 15.80 | 0.00 | 50.00 | 57.14 | 71.43 | 100.00 |
|  |  |  |  |  |  |  |  |
| **(c) Stabilized inverse probability of treatment–weighed sample (n = 325)** | | | | | | | |
|  | **mean** | **SD** | **min** | **Q1** | **median** | **Q3** | **max** |
| **Effectiveness** | 62.89 | 13.92 | 11.11 | 50.00 | 61.11 | 66.67 | 100.00 |
| **Side effects** | 94.81 | 15.00 | 0.00 | 100.00 | 100.00 | 100.00 | 100.00 |
| **Convenience** | 68.32 | 13.57 | 27.78 | 61.11 | 66.67 | 72.22 | 100.00 |
| **Global satisfaction** | 58.06 | 16.19 | 0.00 | 50.00 | 57.14 | 71.43 | 100.00 |
| TSQM, Treatment Satisfaction Questionnaire for Medication; n, number; SD, standard deviation; min, minimum; Q1, lower quartile; Q3, upper quartile; max, maximum | | | | | | | |
